# Governance Experiments in Water Management: From Interests to Building Blocks

**DOI:** 10.1007/s11948-015-9627-3

**Published:** 2015-02-05

**Authors:** Neelke Doorn

**Affiliations:** Department of Technology, Policy and Management - Values, Technology and Innovation, Delft University of Technology, PO Box 5015, 2600 GA Delft, The Netherlands

**Keywords:** Water governance, Stakeholder participation, Governance experiment, Interests, Values, Climate adaptation

## Abstract

The management of water is a topic of great concern. Inadequate management may lead to water scarcity and ecological destruction, but also to an increase of catastrophic floods. With climate change, both water scarcity and the risk of flooding are likely to increase even further in the coming decades. This makes water management currently a highly dynamic field, in which experiments are made with new forms of policy making. In the current paper, a case study is presented in which different interest groups were invited for developing new water policy. The case was innovative in that stakeholders were invited to identify and frame the most urgent water issues, rather than asking them to reflect on possible solutions developed by the water authority itself. The case suggests that stakeholders can participate more effectively if their contribution is focused on underlying competing values rather than conflicting interests.

## Introduction

Climate change is expected to have a significant impact on the availability of water and the frequency and severity of catastrophic floods (CRED 2009; EEA 2010). Although there is a broad consensus that the impact of water scarcity and flooding are increasing in many areas of the world, the conditions under which water scarcity and flooding occurs are still uncertain in several ways. First, the quantitative effect of climate change is still largely uncertain. Though it is by now widely accepted in the scientific community that our climate is subject to change, estimates as to the exact quantitative effects still vary significantly. For example, predictions as to the exact rise in sea level range from approximately 30 cm (lower limit scenario RCP2.6) to 100 cm (upper limit scenario RCP8.5) at the end of the twentyfirst century (IPCC 2013). Similarly, more extreme weather events are expected to occur (both in terms of heavy rainfall and in terms of drought), but these predictions are hard to quantify. Second, demographic conditions may change. Urbanization, for example, may lead to more casualties in cases of coastal flooding. Since these demographic developments are hard to predict with accuracy, so is the related impact. Third, because the nature of the problem and the human role therein is disputed, so is the knowledge base for identifying possible solutions (Driesssen and Van Rijswick 2011). Related to the management of flood risks, some engineers call for traditional (hard) flood protection measures, whereas others opt for “green solutions,” in which agricultural land is “given back to the river.” Together, these uncertainties and ambiguities may influence each other: policy choices are affected by societal and environmental developments and vice versa.

In the terminology of policy science, water management issues could be seen as a typical example of a wicked problem; that is, a problem that is difficult or impossible to solve because of incomplete, contradictory, and changing requirements that are often difficult to pin down. Wicked problems are characterized by ambiguity with regard to the problem definition, uncertainty about the causal relations between the problem and potential solutions, and a wide variety of interests and associated values (Rittel and Webber 1973). The stumbling block does not seem to be an absence of technical solutions but rather the fact that the topic seems to have become too complex and messy to be solved by traditional means (Farrelly and Brown 2011: p. 3). The knowledge and information required to effectively solve these wicked problems are considered to be distributed over different actors. As a result, no single actor has the authority to dominate the process (ibid).

Due to the wickedness of the problem and the fact that knowledge and information are distributed over different actors, people argue for experimenting with different forms of governance to develop an adequate response to the water challenges posed by climate change (Farrelly and Brown 2011; Bos and Brown 2012; Bulkeley and Castan Broto 2013). What these “governance experiments” share is that they put more emphasis on the involvement of stakeholders. For this reason, stakeholder participation is increasingly seen as an indispensable element of water-related policy making, both as a way of democratization (Dryzek 1997; Perhac 1998; Maasen and Weingart 2005) and as a way to improve decision making (Brunner and 2005; Pahl-Wostl 2007; Raadgever and Mostert 2012). However, although supported almost unanimously, it turns out to be difficult to put participation into practice. In the context of water security, stakeholder involvement is part and parcel of the “Integrated Water Resources Management” (IWRM) paradigm, but—although introduced over 40 years ago—the effective implementation of IWRM remains a challenge (Rahaman and Varis 2005).^1^ In Europe, participatory approaches are not as yet systematically included in water governance in a broader sense, although some considerable effort has been made to involve stakeholders in drafting water policy on an experimental basis. For example, quite a number of projects have been initiated by water authorities to ensure the involvement of key stakeholders in the implementation of the Water Framework Directive and the Floods directive. However, these European directives do not fully comply with the international principles concerning IWRM (Rahaman et al. 2004).^2^ Due to the experimental nature, the quality of these stakeholder involvement projects is still largely unknown. Deliberative approaches to stakeholders are sometimes criticized for being biased towards the interests of the most powerful. Attempts to involve stakeholders often reach only those people whose interests are already quite well represented. Similarly, stakeholder involvement may be used to strive for the common good, which is all too easily equated with the interests of the powerful (Mouffe 1999; Young 2000, 2006). Thus, under the sway of deliberation, the appeals to unity and the common good may lead to a bias towards people whose voices are already heard and sideline the legitimized concerns of the most marginalized (Dryzek and Niemeyer 2006), or even worsen their position (McHugh 2006). Another concern is that stakeholder involvement may come at the price of legal certainty and substantive legal provisions before administrative courts (Newig et al. 2014).

This suggests that the involvement of stakeholders is not without risks: it may lead to less rather than more of a voice for the marginalized, and it may lead to less legal certainty and protection (and consequently, less legitimacy), and maybe also less effective policies (Hoffmann 2011: p. 157). In other words, there needs to be an additional normative criterion that makes experimentation warranted. Therefore, the question is when these governance experiments can be considered *responsible* experiments.

In this paper, I present the case study “Deltavision and Water program” to explore the question when and under what conditions governance experiments are likely to be responsible experiments. The Deltavision and Water program is a water project in the Netherlands in which a local water authority involved a wide range of stakeholders to develop a new water policy. I will use the notion of “responsible experimentation” to assess the quality of the decision-making process. The outline of this paper is as follows. Following this introduction, I first elaborate the notion of “responsible experimentation” in Sect. 2, from which I derive a tentative set of criteria for the evaluation of governance experiments. In Sect. 3, I describe the Deltavision and Water program project. In Sect. 4, the project is analyzed in terms of the criteria for responsible experimentation, followed by some concluding remarks in Sect. 5.

Before continuing with my argument, some notes on terminology are in order. In both the literature on governance and the literature on stakeholder participation, a proliferation of meanings can be seen (Van Kersbergen and Verbeek 2004; Avant and Finnemore 2009; Hoffmann 2011).

Kaufmann et al. (1999: p. 1) define governance as “[t]he traditions and institutions by which authority in a country is exercised,” which includes “(1) the process by which governments are selected, monitored and replaced, (2) the capacity of the government to effectively formulate and implement sound policies, and (3) the respect of citizens and the state for the institutions that govern economic and social interactions among them”. Although not literally the same, this definition comes close to the definition of the United Nations Development Programme (UNDP), which defines governance as “the exercise of political and administrative authority at all levels to manage a country’s affairs. It comprises the mechanisms, processes and institutions, through which citizens and groups articulate their interests, exercise their legal rights, meet their obligations and mediate their differences.”^3^ In a 2014 discussion paper on sustainable development, the UNDP states that “[g]overnance is broader than institutions and includes relations between state and people. It provides the mechanisms through which collaboration can be generated across sectors. It also addresses some of the fundamental obstacles to sustainable development including exclusion and inequality” (UNDP 2014).

In the 1997 UNDP document on Governance for Sustainable Human Development, *good* governance is referred to as “[among other things], participatory, transparent and accountable. It is also effective and equitable. And it promotes the rule of law. Good governance ensures that political, social and economic priorities are based on broad consensus in society and that the voices of the poorest and the most vulnerable are heard in decision-making over the allocation of development resources” (UNDP 1997).

Specific to the water domain, it is recognized that water governance involves different administrative scales and spatial dimensions, which is usually referred to as multi-level governance. Although a one-size-fits-all approach is not suitable, it is possible to formulate a set of criteria that indicate the degree to which water governance can be considered good water governance (Toonen and Doorn 2015). The OECD identified the following set of universal aspects of good water governance (OECD 2011, 2014):

- legitimacy in complying with international and European Union requirements;
- subsidiarity in performing tasks allocated in the framework of a decentralized unitary state;
- effectiveness in delivering policy outcomes in a transparent way and achieving expected results;
- efficiency in doing it at the least cost;
- equity in ensuring fairness in the service delivery and allocation of uses.

In this paper, I will follow the widely accepted descriptions provided by the UNDP and the OECD. By governance I refer to the exercise of political and administrative authority at all levels to manage a country’s affairs, comprising the mechanisms, processes and institutions, at all administrative levels, through which citizens and groups articulate their interests, exercise their legal rights, meet their obligations and mediate their differences, including the relations between state and people. By experiment, I refer to those acts, plans, or projects in which one deliberately does something new, or does something in a new way with the aim to improve the quality but with an uncertain outcome. Governance experiments can then be conceived of as deliberate attempts to exercise political and administrative authority in a hitherto unconventional way with the aim to improve the legitimacy, subsidiarity, effectiveness, efficiency, and equity of the mechanisms, processes and institutions through which citizens and groups articulate their interests, exercise their legal rights, meet their obligations and mediate their differences, including the relations between state and people.

All attempts in which stakeholders get a new role they had hitherto not fulfilled, carry some degree of uncertainty and, as such, are experimental. However, not all types of governance involve the participation of stakeholders. I therefore view the projects aimed at stakeholder involvement as a subset of the larger set of governance experiments. Stakeholder participation and stakeholder involvement are seen as synonymous.

## A Framework for Experimentation

Since the last decade, the notion of experiment or experimentation has popped up in different bodies of literature, ranging from risk governance and policy science (Krohn and Weyer 1994; Herbold 1995; Hoogma 2002; Gieryn 2006; Gross 2010), to engineering and risk ethics (Martin and Schinzinger 2005; Van de Poel 2009; Jacobs and Van de Poel 2010), and transition management (Hoogma 2002; Raven and Van den Bosch 2007; Van der Brugge and Rotmans 2007).

Dependent on the context, the use of the notion of experimentation has a different function. In debates about the acceptability of risky technologies, the notion of experimentation may have a pejorative meaning, referring to the experimental subject being used as a “guinea pig” (Felt and Wynne 2007: p. 68). In governance and policy literature, on the contrary, experimentation is often seen as an adequate response to wicked problems, because experiments allow for the inclusion of stakeholders, which in turn may prompt learning processes that are impossible to achieve by other means (OECD 2014). Most of these experiments are therefore shaped and evaluated in terms of the degree of learning experienced within the network or organization of relevant actors (mostly referred to as collective, organizational, or social learning to distinguish it from learning that occurs on an individual basis). However, despite the support for social learning, there is limited scientific understanding of how and to what extent the participation of stakeholders actually generates these learning processes (Reed and Evely 2010). Consequently, the added value of governance experiments is not clear-cut. It is therefore important to develop criteria for when a governance experiment can be deemed responsible and, as a next step, to see under what conditions those criteria are most likely met.

The set of criteria for when an experiment can be deemed responsible can be based on the description of good governance, given in the previous section. In that section, I referred to good governance as mechanisms, processes, and institutions that confirm the criteria of legitimacy, subsidiarity, effectiveness, efficiency, and equity. Based on these criteria, a responsible governance experiment is an experiment that has a positive contribution to one or more of these criteria without reducing the performance on any of the other criteria (Pareto-improvement).

The question under what conditions these criteria are most likely met is an empirical question. The framework that I will use to describe these conditions is based on a recent study by Hoffmann (2011) on climate governance experiments. Based on an analysis of 58 experiments, Hoffmann identified four experimental governance models. The four models are the networkers (model 1), the infrastructure builders (model 2), the voluntary actors (model 3), and the accountable actors (model 4). Hoffmann analyzed these models in the context of climate governance and primarily in the mitigation of climate change. Each model has one or more core functions to fulfill in responding to climate change (Hoffman, p. 34):

1. The *networking function* is primarily aimed at information diffusion. It covers activities like education, information exchange, sharing best practices, and regular meetings. The fundamental assumption behind this function is that the main problem with the response to climate change is poorly distributed information and resources to take action and not so much lack of will. The solution is therefore to “bring actors together—physically and/or virtually—to spread ideas and practices that can be implemented. The goal of networking is to catalyze action outside the jurisdiction of the experiment itself through peer-to-peer motivation and learning” (Hoffmann, p. 43).
2. The *planning function* covers activities that prepare actors for taking actions and to facilitate the global response to climate change. Typical activities are developing measuring techniques and protocols to track carbon emissions, creating action plans and certification standards, and undertaking risk assessments (Hoffmann, p. 34).
3. The *direct action function* covers efforts to directly reduce greenhouse gas emissions, which includes efficiency measures, offset programs, rationing, emission trading, and technological development (Hoffmann, p. 34).
4. The *oversight function* covers activities that are aimed at monitoring and enforcing the commitment of implementing actors.

Table 1 shows how the four functions relate to the four models. While the first two models are primarily aimed at building the foundations for more tangible action (either by networking or by planning), the third (voluntary actors) and fourth (accountable actors) models aim at prompting actions to reduce greenhouse gas emissions, in addition to the networking and planning functions. The experiments in the third model lack formal accountability structures. Since the actors in the third model often share a common ethos, enforcement is not required to achieve compliance. As Hoffmann explains, “many of the voluntary action experiments are undertaking action not so as to avoid mandatory regulations but so as to go beyond existing regulations or as a way to press for more stringent regulation” (p. 51). The overall goal of this third model could therefore also be seen as setting an example. Examples of this type of experiments are initiatives to implement green building standards, travel offsets, and waste reduction programs. The accountability model, lastly, is the most demanding model, including all three activities of the other three models (networking, planning, and direct action) with the existence of “oversight measures that make the action accountable” (p. 55). Interestingly, the experiments studied by Hoffmann indicate that moving from voluntary to mandatory action does not require the presence of governmental authority. Also, outside the governmental realm, accountability schemes can be introduced to enforce the commitment of implementing actors, for example, by penalties for not staying within one’s carbon emission allowance.

**Table 1:** Governance models and core functions

Model	Core function			
	Networking	Planning	Direct action	Oversight
Model 1: The networkers	X			
Model 2: The infrastructure builders		X		
Model 3: The voluntary actors	X	X	X	
Model 4: Accountable actors	X	X	X	X

Hoffmann claims that a balanced mixture of all types of experiments, or at least all functions, is required for developing an efficient and effective response to climate change. His main concern is that actors will participate only in those experiments that serve their own interests. This may result in a situation that “experimentation will not catalyze broader change” (p. 77). His preliminary answer to this problem is the recognition that there is an experimental *system* of governance. Experiments that are currently on separate paths need to be brought together so that different experiments can learn from each other. At the same time, the experimental system of governance needs to be aligned with the existing multilateral treaty-making (an activity that is especially relevant in the context of climate change). Multilateral treaty-making can then be used to enhance innovations in governance experiments. Hoffmann argues for leadership, both within the experimental system and from the traditional multilateral governance system. Hoffman’s background assumption is that, as long as there is sufficient uncertainty, experiments will follow. Leadership from within the experimental system is therefore about sustaining this uncertainty and creating friction. Leadership from the traditional multilateral governance system “is a matter of restraining ambitions for a comprehensive global treaty and instead using treaty-making tools to enhance the burgeoning activities already taking place—a global framework signaling that climate will be addressed seriously along with agreements to regulate specific activities” (p. 162).

Against the background of the book and Hoffmann’s background in global environmental politics, Hoffmann’s plea for taking a systems approach to experimental governance is certainly not surprising. However, this focus on the system level does not provide much guidance for actors trying to set-up specific governance experiments. Also *within* specific experiments, Hoffmann’s fear of people merely pursuing their own interests may be present. The popular narrative in the literature on participatory approaches is indeed one in which parties fight over competing interests. Environmental conflicts, for example, are often framed as conflicts between pro-environmentalist and anti-environmentalist positions, with the latter pursuing their own self-interests (Sagoff 2002; Kline 2007). For a private organization this does not need to be problematic, but for a governmental body trying to initiate governance experiments, this may be a severe problem. There is a growing body of literature which indicates that environmental conflicts might more accurately be framed as conflicts over competing ethical frameworks (CAST 2005; Glenna 2010), and that failing to address the underlying ethical issues may even create and exacerbate conflict (Rikoon and Goedeke 2000; Wilshusen and Brechin 2003). In other words, it is certainly not the case that “it doesn’t hurt to try.” The set-up of governance experiments *on behalf of a governmental body* (in the remainder referred to as: official governance experiment) should be carefully chosen to prevent people from merely pursuing their own self-interest, which can be deemed bad governance. Following Hoffmann’s empirical work, this can be prevented—and even stronger: the opposite will probably be achieved—in case all functions are fulfilled.

I therefore propose to use Hoffmann’s functions as a tentative set of conditions under which it is likely that governance experiments lead to good governance. My hypothesis is that an official governance experiment will contribute to good governance if the following four functions are fulfilled:

1. a networking function aimed at information diffusion;
2. a planning function aimed at facilitating actions;
3. a direct action function;
4. an oversight function aimed at monitoring and enforcing the commitment to implementing actors.

In the remainder of this article I will explore this set of functions further by discussing a governance experiment in the Netherlands. The case study concerns the development of a water policy plan for the period 2016–2021. Hoffmann’s analysis concerned experimental governance systems aimed at climate change mitigation. In the context of water governance, climate change itself is more or less a given and the question is how to respond to the impact of climate change. The current debate in water governance is therefore more about *adaptation to* than about *mitigation of* climate change. Yet, adaptation and mitigation pose similar governance challenges: the distribution of responsibilities, competing interests, and value conflicts. The reason for turning to governance experiments in climate adaptation is exactly because of the “wicked” nature of the problems. This is shared by both climate mitigation and climate adaptation experiments.

One caveat is due at this point. My view on experimentation is different than Hoffmann’s view. Hoffmann seems to claim that experimentation is good as long as it fulfils the functions elaborated above (Hoffmann 2011, p. 102). Consequently, insecurity and especially uncertainty are good because this may prompt even more experimentation. I am more sceptical about experiments and especially about the loss of legal protection and legal security. I agree that wicked problems require the involvement of a wider range of actors than the usual ones in the policy making process. However, this is only to be welcomed if it does not come at the expense of legitimacy. I therefore conceive of experimentation not as a goal in itself but as instrumental to good governance. In line with this, the functions identified by Hoffmann are not seen as criteria but rather as a set of conditions that describe under which circumstances governance experiments are likely to contribute to good governance.

## Case Study: The HHNK Deltavision and Water Program

The experiment that I will discuss in this paper is the water program developed by the *Hoogheemraadschap Hollands Noorderkwartier* (HHNK), one of the water boards in the Netherlands.^4^ The HHNK covers an area in the north-western part of the Netherlands. Before discussing the case study itself, it is important to know something more about the geographical context of the case study. The Netherlands is low-lying delta in north-west Europe with a relatively high population density. With approximately 20 % of the country below sea level, 50 % <1 m above sea level, and two-thirds of the population living in flood-prone areas, the Netherlands is extremely vulnerable to flooding. Additionally, four large transboundary rivers—the Meuse, the Rhine, the Ems, and the Scheldt—flow through the Netherlands into the North Sea, making the country also vulnerable to riverine flooding. Without protective measures, more than half of the country is threatened by flooding from either land or sea (Van Rijswick and Havekes 2012). It is therefore not surprising to see that water management has always played an important role in the Netherlands.

Water management in the Netherlands is mostly organized at the local and regional level. The Dutch regional water authorities, the water boards, are among the oldest forms of local government in the Netherlands. The history of some water boards dates back to as early as the thirteenth century (Bulkeley and Castan Broto 2013). Up to the end of the 20th century water legislation was heavily fragmented. There was a need for a more integrated legal framework for safety against flooding, water quality and water quantity. The same need for integration was seen at the European level, where various international initiatives had been launched with the aim of achieving more coherent and integrated water policy (Van Rijswick and Havekes 2012). The most important outcome of this process is the Water Framework Directive (WFD; Directive 2000/60/EC) aimed at achieving a “good status” of all bodies of water. For the Netherlands, the European Floods Directive (Directive 2007/60/EC) is also particularly important.

The need for integrated water legislation and the influence of European legislation resulted in the Dutch Water Law, which came into force in 2009. The traditional decentralized nature of Dutch water management is also visible in this new Water Law, though the European directives had some centralizing effect on the Dutch Water Law (Van Rijswick and Havekes 2012).

Parallel to the introduction of the new Water Law, the Dutch system of safety norms is being reviewed in 2014–2015. In the Netherlands, safety against flooding is laid down in law through a system of safety norms that distinguishes between different so-called dike-ring areas. A dike-ring is an uninterrupted ring of water defenses, which, together with high grounds, protects the area within the dike-ring against flooding. The safety norm for each dike-ring is dependent on the economic value of the dike-ring area. Although this allows for some differentiation in safety norms within the jurisdiction of one water board, until 2014 most areas within the jurisdiction of one water board are protected at approximately the same level.

In 2007, a ministerial advisory committee (the “Delta committee”) was installed with the task of advising the government on how to develop a long-term vision on flood safety for the Netherlands, taking into account climate change. In its advice in 2008, the committee argued for a new system of safety norms. The old system of safety norms, which was still based on economic calculations from the 1950s, proved outdated. It also appeared that a large number of flood defenses did not meet the statutory levels of safety. The new system of safety norms has more differentiation in safety levels, also within single dike-ring areas. This new system will be put into place in 2014–2015. From then on, the water boards will also be required to contribute financially to the implementation of flood protection measures, which was until 2014 a national concern, based on the solidarity principle and shared equally by all citizens. With the new payment system, safety against flooding is primarily determined on the basis of how densely populated the area under jurisdiction of the water board is and the length and number of dikes to be improved or maintained by the water boards.

These developments prompted some water boards to develop their own water plans on an experimental basis. Especially the issue of norm differentiation *within* one dike-ring area prompted substantial resistance among the people working for the water boards. Many water boards struggled with the question how to justify to citizens in the same area that some are protected at a higher level than others?

The project “Deltavision and Water program” of the water board HHNK is currently one of the hallmark success projects in the Dutch water sector that does indeed try to address this issue. The water board is praised for its innovative approach to actively involve stakeholders in the development of water policy. In addition to climate change and the water policy developments at the national level, HHNK mentioned the ambition to carry out its task in cooperation with citizens, interests groups and other governmental bodies as a direct occasion for the Water program. The water board justified this choice based on reasons of both efficiency and efficacy. They expected that cooperation with other parties would, in the end, lead to better solutions against lower societal costs.

The starting point for the Water program is the Deltavision, developed by the water board. The Deltavision is the water board’s long-term strategic vision on water policy, presented in terms of three themes: water safety, water nuisance, and sufficient clean freshwater. The aim of the Water program is to develop an intermediate-term water policy plan for the period 2016–2021, which includes the identification and prioritization of necessary measures.

The combined process of drafting the Deltavision and Water program consists of several, partly iterative, phases.

1. Fall 2011-fall 2012: The Deltavision was formulated in cooperation with seven societal partners: representatives from the nature conservation sector, representatives from the agricultural sector, regional authorities, the local drinking water company PWN,^5^ knowledge institutes, Rijkswaterstaat (the national executive agency for public works), and the water board itself. In the remainder of this text, these seven partners are referred to as “the societal partners”. Based on an analysis of climate trends and scenarios and the current status of the water system, dilemmas for each of the themes were identified, which resulted in a list of 13 dilemmas. These dilemmas were further elaborated and presented as brief video clips on the water board’s website. The dilemmas served as a starting point for discussing the board’s water policy with the seven societal partners. In this consultation round, it was deliberately chosen not to search for solutions but to focus on the formulation of the dilemmas first. For this reason, the consultation sessions were organized with a single partner (that is, with one of the seven partners listed above) so that there was no reason to negotiate particular interests. During these sessions, some dilemmas were combined and some were reformulated. After this first consultation round, a preliminary version of the Deltavision was drafted, which was again discussed in a series of working sessions with the societal partners. A widely supported Deltavision was the result of these two consultation rounds. The Deltavision is available online on the water board’s website.
2. 2013: The second phase was to move from a strategic Deltavision to the development of an action guiding water program. In order to do this, a series of “water table meetings” were organized. These meetings were again dedicated to one societal partner per meeting. In addition to the seven mentioned above, some new partners were identified: water-related companies, non-water-related companies, fisheries, cable and pipe companies, and one general water table meeting, in which individual people could participate. During these meetings, over 300 people provided input. Participants were explicitly asked whether they wanted to develop solutions and in cooperation with which partner they wanted to do this. The organizers of these meetings collected over 1,000 quotes about the three themes water safety, water nuisance, and sufficient clean fresh water.
3. Winter-spring 2014: The input collected during the “water table meetings” led to the formulation of 35 building blocks by the water board. These building blocks referred to potential topics on which different partners wanted to cooperate. In a meeting with representatives of all societal partners, the building blocks were discussed and some of them were reformulated or combined with other building blocks. Examples of these building blocks included: nature as a solution for (additional) safety, surface water without traces of hormones and drugs, combining water functions, multifunctional water defenses as a storage place for cables and pipes. In a joint-partner meeting with representatives of all societal partners involved, the societal partners were asked to select two building blocks that they wanted to cooperate on. For each of the selected building blocks one responsible partner was identified and one sponsor. This led to the formation of 16 building teams.
4. Spring 2014: After formation of the building teams, the Water program itself was developed. This Water program is a concrete action guiding water policy plan for the period 2016–2021. As explained, some of the input collected during the water table and other meetings led to the formulation of building blocks and, after that, building teams. However, a lot of the input collected during these meetings fell outside the scope of the building blocks because it belonged to the core tasks of the water board itself and could not be delegated to the societal partners. This information was interpreted as an important signal to be included in the Water program directly. Hence, the Water program will eventually consist of a part developed by the water board itself, and informed by the several stakeholder meetings, and a number of building blocks developed by the societal partners. The first draft of the Water program, including first results of the building teams, was discussed in a meeting with almost 200 people, representing 70 organizations.
5. Fall 2014-winter 2015: The resulting Water program will be presented to the executives of the water board, and after approval, the Water program will be made available for public access following the regular legal administrative procedures.

**Fig. 1:**
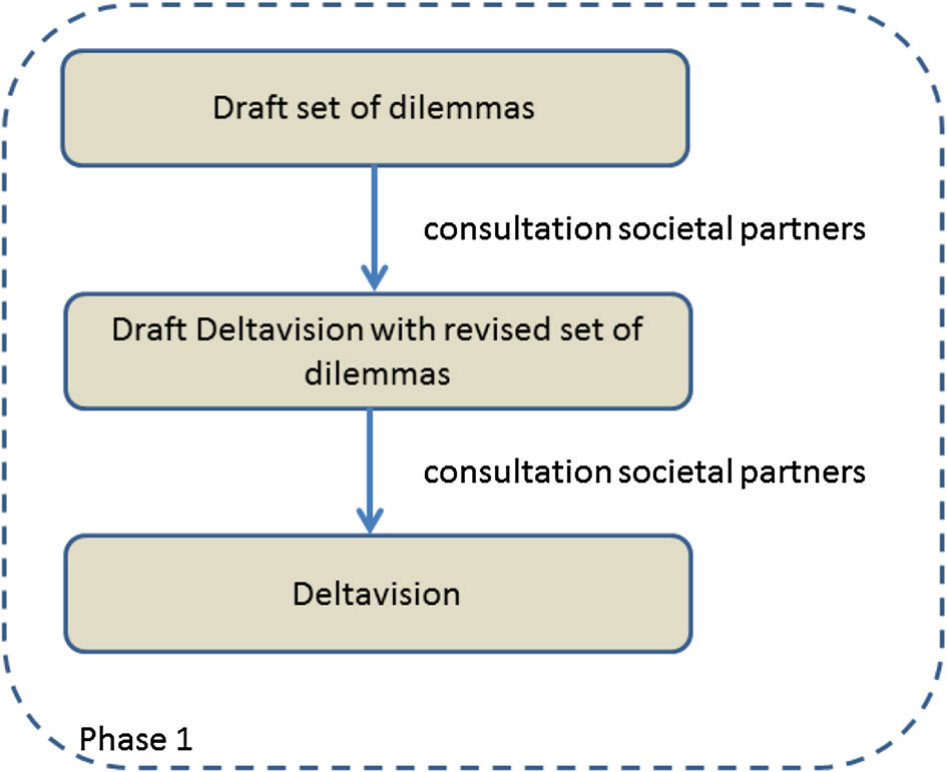
Formulation of the Deltavision

**Fig. 2:**
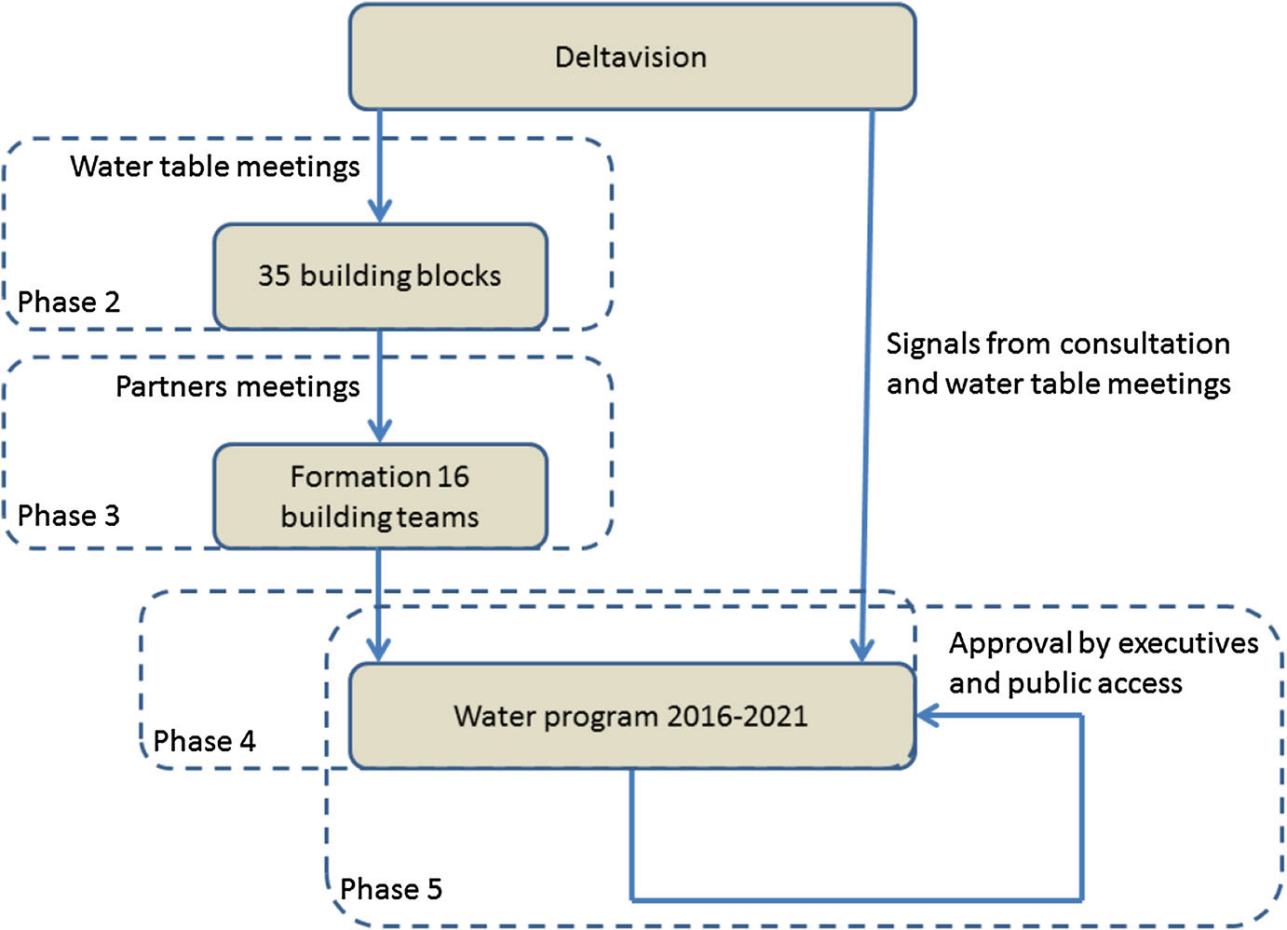
Development of the Water program 2016–2021

Figures 1 and 2 show the different phases in the formulation of the Deltavision and the Water program respectively.

## Discussion

If we look at the respective phases in the development of the Deltavision and the Water program, we can recognize the different functions described by Hoffman (see also Table 2 for an overview of the different phases and the core functions in each phase).

**Table 2:** Phases in the project and core functions

Phase	Core function			
	Net-working	Planning	Direct action	Oversight
Phase 1: Formulation Deltavision	X			
Phase 2: Identification building blocks	X	(X)		
Phase 3: Formation building teams		X		
Phase 4: Formulation draft water program			X	
Phase 5: Approval and public access				X

The core function in phase 1 (the development of the Deltavision) is the networking function. In this phase, information was gathered from the different societal partners and the different societal partners were informed about the activities by the water board. The aim of this phase is not to exchange information between different societal actors. It is also important to note that this phase is not aimed at developing solutions, which would already go into the direction of the planning function, but rather at formulating the main challenges.

The core function of phase 2 (the identification of 35 building blocks) is again the networking function. Like in phase 1, the aim is to exchange information, but in phase 2, the networking function goes beyond the definition of the problem and already focuses on possible solutions. This is most visible in the identification of the building blocks. These building blocks can be seen as possible solution directions to challenges identified in the Deltavision itself. The formulation of the building blocks can also be seen as a first example of the planning function. The building blocks lay the foundation and provide tools for developing a response to the water challenges. Similar to phase 1, the societal partners are not yet brought together.

The core function of phase 3 is the planning function. As mentioned, the formulation of the 35 building blocks already preluded at this planning function, but this function gets really substantiated in phase 3 in which the building teams are composed. Phase 3 is explicitly designed to prepare the partners for taking action. It is also the first phase in which different societal partners are brought together to develop plans.

In phase 4, stakeholders representing different societal partners work together on the formulation of the draft Water program. This could be seen as a form of direct action, though still on a voluntary basis. There are no means of accountability to secure the commitment of the building teams. However, the water board explicitly tried to get commitment by asking the societal partners to develop their own plans (in the sense of giving input to the water program) rather than merely asking their feedback on proposals developed by the water board itself.

Phase 5, lastly, represents the oversight function. The Water program obtains juridical force after approval by the executives of the water board and the formal administrative public access procedures. The approval by the executives of the water board is especially important as a kind of “reality check”: without this approval the Water program would remain a nice and ambitious, but as yet fruitless attempt to involve stakeholders. In the end, the water board is a formal administrative body that is responsible for water safety and water security in the area. Before some plan can count as the official policy of this administrative body, the plan needs to have followed the official legal procedures. In this phase, budgets will also be allocated to the plans of the different building teams.

At the time of writing, the Deltavision and the Water program are considered a great success in the Dutch water field. Opponents of technocratic top-down decision making praise the attempts of the HHNK for their genuine effort at giving stakeholders a role in the development of water policy. Skeptics about stakeholder participation praise the water board for their success in getting and sustaining the commitment of such a large group of stakeholders. Between phase 4 and 5, the Deltavision and the Water program evolved into an ongoing process of stakeholder involvement with an outlook beyond the actual completion of the Water program. Assessed in terms of the criteria of good governance (legitimacy, subsidiarity, effectiveness, efficiency, and equity) the project can indeed be seen as an example of good governance. The involvement of the stakeholders has certainly increased the legitimacy and subsidiarity of the project. Because people have been able to voice their concerns and because the stakeholders have committed themselves to the project, the whole process has probably also contributed to equity and effectiveness. Assessment in terms of efficiency is difficult, and the answer may be different if we look at the short-term achievements and the long-term achievements. Involving the public may, in the long term, be more efficient but a follow-up study would be necessary to empirically justify this claim. The water board itself indicated that they profited from relations built in previous projects, which suggests that the efficiency gain of these kinds of projects may also extend to future projects.

As mentioned in Sect. 2, the framework of Hoffmann was used to identify the conditions under which governance experiments could most likely be deemed good or responsible. The discussion shows that all four function identified by Hoffmann were indeed clearly present in the project. However, this does not prove that this set of functions is either necessary or sufficient for developing responsible governance experiments. Yet, the formulation of the Deltavision and the development of the Water program have some striking elements that could possibly explain their success and these elements do refer to the functions identified by Hoffmann.

The first concerns the timing of the different phases and the moment of exchanging information between stakeholders representing different “interest groups.” In the phase of developing the Deltavision, the water board has put considerable effort in formulating the problem and dilemmas. They tried not to search for solutions at too early a phase, but rather to come to a formulation of the challenges in which all stakeholders recognized themselves. In what followed, stakeholders were given a role in developing solutions. It has been recognized for quite some time now that the way a problem is framed will also determine the kind of solutions people see as being in their interest (Schön and Rein 1994). However, few stakeholder involvement projects give the stakeholders such a profound role in both the formulation of the problem and the development of solutions. The project leaders of the Deltavision and the Water program indicated that, as long as they were exploring the problem, they explicitly aimed at meetings with representatives of one single societal partner because that would allow them to discuss the possible problem definitions without going having to negotiate these with other “interest groups.” Based on experiences with previous stakeholder projects, they expected that these meetings with single societal partners would also allow them to discuss the problem definition more in-depth and to focus on underlying values rather than interests. A meeting attended by different societal partners would run the danger of searching for solutions before the problem was adequately explored because people could possibly feel that they had to protect their own interests. As such, the initial experimentation space was a protected space in which stakeholders could give their input without having to negotiate their interests. It was only once the societal partners had committed themselves to the project and once the dilemmas were formulated, that the water board organized meetings in which representatives from different societal partners were present at the same meeting. The timing of the different phases also proved productive in terms of securing commitment from the different stakeholders. Empirical research in political theory has shown that stakeholder involvement may conflict with the ideal of deliberation (Mutz 2006; Doorn 2013). Hence, it may be good to postpone the deliberation between people representing different societal groups to a phase when the stakeholders have already committed themselves to the project. In the current project, commitment was created in the first two phases. Deliberation with representatives from other societal groups started only in the third phase. These phases correspond to the order of the functions identified by Hoffmann.

A second striking element in the development of the water program was the way in which the people from the water board obtained the commitment of both the societal partners and the executives from the water board. The number of people attending the different meetings (almost 200) and the number of organizations represented (70) is impressive if one realizes that the awareness of water related policy making is notoriously low in the Netherlands (Heems and Kothuis 2012; see also the recent report by the OECD on water governance in the Netherlands, OECD 2014). The project leaders explicitly labeled the Deltavision and the Water program an experiment with a—for them—uncertain outcome. It was new for them to ask societal partners not only to give feedback on policy plans developed by the water board but to let them develop elements of policy plans themselves. However, some stakeholders indicated that the overall process was time consuming, which may jeopardize their commitment in the long run. It is important that the involvement of stakeholders has a visible impact on the Water program and that the concrete result goes beyond the fulfilment of the democratic ideal of “having been able to have a voice in the process.” This suggests that Hoffmann’s networking and planning function is important, but that these functions do not suffice. In the end, the involvement of stakeholders should lead to accountable action.

A third important element is that by securing the approval of the executives of the water board and by following the usual legal procedures of public access, the Water plan gets full legal status. Since stakeholder projects may sometimes suffer from a lack of legal certainty and protection, the commitment of the board’s executives contributes to the legitimacy of the project. By securing both the commitment of the higher executives of the water board and of the societal partners, the project was able to avoid the pitfalls of a strictly top-down and a strictly bottom-up approach. Again, this points to the importance of the oversight function in Hoffmann’s framework.

One question that remains open is the level of concreteness that the building teams are able to achieve. It is the aim that the elaborated building blocks will become part of the water board’s Water program, which aims to be a concrete policy document for the intermediate term. If the building blocks elaborated by the building teams are to have an impact on the water board’s policy, they should have some minimal level concreteness. At the time of writing, it is still open whether this will indeed be achieved. Getting agreement on very general points is sometimes considered to be more easily achievable than agreement on concrete decisions. However, it may be the other way around as well. People may, for example, agree on concrete points but disagree on the underlying values (Doorn 2010). In those situations, it may equally well be that people agree on concrete decisions, be it for different reasons (Doorn 2012). The fact that after presentation of their initial plans in spring 2014, the building teams were asked to develop budgets suggests that the teams were indeed able to achieve some level of concreteness.

## Concluding Remarks

In this paper, I discussed a water governance experiment in the Netherlands to illustrate that governance experiments can contribute to *good* governance. Based on the UNDP and OECD definitions of good governance, I used the governance experimentation framework, developed by Hoffmann (2011), to explore under what conditions these experiments lead to good governance. Contrary to Hoffmann’s own use, the framework is not used as a normative theory to discuss good governance, but rather as a set of conditions under which official governance experiments are likely to contribute to good governance.

Three tentative lessons could be drawn from this case study.

The first lesson is that effort should be put into preventing these stakeholder projects from becoming negotiations. If the involvement of stakeholders becomes a negotiation process between people with conflicting interests, this may jeopardize the position of minority groups. In this project, this was prevented by paying due attention to the definition of the main challenges. Representatives of the same societal groups discussed the challenges in a protected environment in which they did not have to stand up for their own position. This prompted a discussion in terms of important values and ideals rather than in terms of conflicting interests, which proves much more constructive for finding solutions at some later stage (cf. Glenna 2010).

The second lesson concerns the commitment of the societal partners. The project suggests that it may be good to postpone deliberation between people representing different societal groups to a phase when the stakeholders have already committed themselves to the project. In the current project, commitment was created in the first two phases. Deliberation with representatives from other societal groups started only in the third phase.

A last lesson concerns the limits of participation. Governance requires a subtle balance between top-down decision making and bottom-up approaches. It is important that policy that is drafted by or in cooperation with stakeholders is also supported by the executives of the responsible administrative body.

Further research is needed for exploring the full potential of governance experiments. The case study discussed in this paper suggests that it is possible to organize governance experiments without loss of legitimacy if these experiments fulfill the functions of networking, planning, direction action, and oversight. More research is needed to see whether these are necessary or sufficient conditions for responsible experimentation and, in case of the latter, what the alternative routes are to responsible experimentation.

## Footnotes

1 See Hassing et al. 2009 or the website of the Global Water Partnership (http://www.gwp.org/en/The-Challenge/What-is-IWRM/) for an overview of IWRM in practice.

2 E.g., the UK (Woods 2008; Nye et al. 2011), Germany (Heintz et al. 2012), Italy (Soncini-Sessa 2007); see also Warner et al. (2013) for a comprehensive discussion.

3 Committee of experts on public administration, definition of basic concepts and terminologies in governance and public administration (E/C.16/2006/4) (New York, 2006).

4 Relevant data of the Water program can be found at http://www.hhnk.nl/deltavisie/deltavisie/waterprogramma (accessed June 30, 2014), including reports, minutes of meetings, and interview data.

5 In the Netherlands, drinking water companies that retrieve their water partly from natural ground water resources also have a nature conservation task. This holds for the PWN as well.
